# Cellular angiofibroma of the vagina: A case report and literature review

**DOI:** 10.1097/MD.0000000000030293

**Published:** 2022-09-02

**Authors:** Xia-Qin Cai, Xi-Gang Ye, Ya-Zhen Zhang, Zeng-Li Shen, Ke Hong, Shu-Zhi Zhang

**Affiliations:** a Department of Obstetrics and Gynecology, Tongde Hospital of Zhejiang Province, Hangzhou, Zhejiang, China; b Sanmen People’s Hospital of Zhejiang Province, Sanmen, Zhejiang, China; c Hangzhou Gongshu District Wenhui Street Community Health Service Center, Hangzhou, Zhejiang, China; d The Pathology Department, Tongde Hospital of Zhejiang Province, Hangzhou, Zhejiang, China; e Hangzhou Medical College, Hangzhou, Zhejiang, China.

**Keywords:** case report, cellular angiofibroma, immunohistochemistry, pathology, vagina

## Abstract

Cellular angiofibroma (CAF), a rare benign mesenchymal tumor, is histologically characterized by abundant thick-walled vessels with a spindle cell component. As one of the female reproductive system tumors, its clinical and pathological features are not well characterized.

**Methods:**

A 47-year-old woman presented for the removal of intrauterine device on October 28, 2021, as she had achieved menopause one year back. The patient had no discomfort or awareness of any mass in her vagina. She has history of breast cancer and papillary thyroid cancer. Till date, no progression of thyroid cancer or breast cancer has been observed. Her menstrual cycle was regular, and she had one child delivered vaginally.

**Results:**

Pelvic examination revealed a mass sized 2.5 × 2.0 cm located near the fornix in the upper segment of the left vaginal wall. Thin prep cytologic test (TCT) revealed negative intraepithelial lesion or malignancy (NILM). HPV test was negative and leucorrhea routine inspection cleanliness II degree. No cervical mass was detected by ultrasound examination. The patients underwent the operation for intrauterine device removal plus vaginal tumor resection on November 1, 2021. Postoperative antibiotics (intravenous cefuroxime sodium 0.75 g bid for 1 day) were administered to prevent infection. The patient showed no signs of recurrence at one-month follow-up.

**Conclusion:**

In summary, CAF is a rare benign soft tissue tumor. Surgery is the only treatment method, and the definitive diagnosis of CAF is based on histopathological examination of surgical specimen. Long-term follow-up is needed for surveillance of recurrence.

## 1. Introduction

Cellular angiofibroma (CAF), a rare benign mesenchymal tumor, is histologically characterized by abundant thick-walled vessels with a spindle cell component.^[1]^ CAF occurs in both men (referred to as “angiomyofibroblastoma like tumor”) and women (particularly in the vulva).^[2]^ Due to its low incidence, articles pertaining to vulvar CAF in the published literature largely comprise of small case series or case reports.^[2–13]^ Owing to the non-specific clinical manifestations of CAF, preoperative diagnosis is typically challenging. Pathological examination is required to confirm the diagnosis of the disease. According to a recent review, only a few cases of vaginal CAF have been reported.^[14]^ Although the World Health Organization (WHO) has classified CAF as one of the female reproductive system tumors, its clinical and pathological features are not well characterized.^[15]^ We report a case of vaginal CAF treated by surgery.

## 2. Case report

A 47-year-old woman presented for the removal of intrauterine device on October 28, 2021, as she had achieved menopause 1 year back. The patient’s last menstruation was on October 10, 2020. She had been intermittently using the intrauterine device for 22 years. The patient had no discomfort or awareness of any mass in her vagina.

She was diagnosed with invasive breast cancer in right breast and had undergone breast preservation surgery plus axillary dissection on February 2, 2016. The size of the excised tumor was 0.8 × 0.6 × 1.8 cm. The subsequent systematic therapy included 29 cycles of postoperative radiotherapy and 6 cycles of chemotherapy. She has continued to receive tamoxifen and has been regularly followed-up at the Shanghai Fudan University Cancer Hospital for more than 5 years since the operation. There was no recurrence of breast cancer. The patient also underwent a thyroid biopsy on May 17, 2017, due to long-standing thyroid nodules for 30 years. The histopathological diagnosis was papillary thyroid cancer. She underwent surgery for the removal of unilateral thyroid gland on August 22, 2020. She was then prescribed levothyroxine tablet (50 µg) once daily. Till date, no progression of thyroid cancer has been observed. Her menstrual cycle was regular, and she had delivered 1 child vaginally.

## 3. Clinical findings and diagnostic assessment

Pelvic examination revealed a mass sized 2.5 × 2.0 cm located near the fornix in the upper segment of the left vaginal wall. The mass was firm in texture with a thick pedicle, approximately round contour, non-tender, and flexible mobility. The cervix was normal in size with mild erosion. The uterus was slightly enlarged and retroverted but showed no tenderness. The uterine appendages were normal. Based on the pelvic exam, a diagnosis of vaginal wall cyst was considered.

Thinprep cytologic test (TCT) revealed negative intraepithelial lesion or malignancy (NILM). HPV test was negative and, on leucorrhea routine inspection, the cleanliness was rated as degree II. No cervical mass was detected by ultrasound examination.

## 4. Therapeutic intervention

After admission, the routine preoperative investigations (including routine blood tests, coagulation function, biochemistry, thyroid function, and electrocardiogram) showed no abnormality. She then underwent the operation for intrauterine device removal plus vaginal tumor resection on November 1, 2021. During the surgery, the root of the tumor was clamped with 2 vascular clamps, and tumor was completely removed. The surgical wound was sutured with a 2-0 absorbable line in the shape of “8.” After the operation, 2 pieces of PVP gauze were filled in the vagina to prevent bleeding and were taken out after 24 hours of the operation. Postoperative antibiotics (intravenous cefuroxime sodium 0.75 g bid for 1 day) were administered to prevent infection.

## 5. Follow-up and outcomes

**Figure 1.**
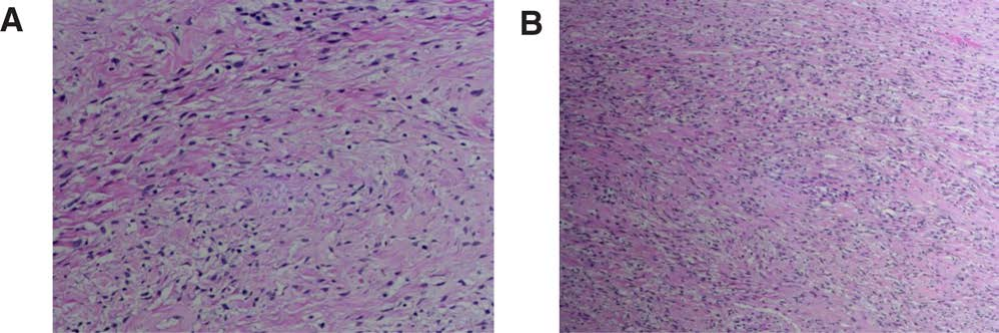
H&E-stained sections of the surgical specimen. The tumor tissue is composed of short spindle cells and large blood vessels. (A) ×400 magnification. (B) ×100 magnification.

Pathological examination of the surgical specimen was performed. The tumor was 2.5 cm in diameter, round, with a smooth surface, clear boundary, and visible capsule. Cut section of the tumor revealed gray and white areas with solid texture, and no signs of necrosis. Microscopic examination of the HE-stained sections showed that the tumor tissue was composed of short spindle cells and large blood vessels (Fig. 1). The cells were arranged in a disorderly manner with areas of hypercellularity interspersed with hypocellular areas. The nucleus of tumor cells was oval, with scant cytoplasm and unclear borders, and occasional mitotic figures. There were abundant small or medium-sized blood vessels with variable thickness of vessel wall. In addition, areas of hyaline degeneration, interstitial focal edema, and mucinous degeneration were found in parts of the blood vessel wall. The pathological diagnosis of the vaginal mass was spindle cell tumor.

Immunohistochemistry examination showed diffuse positive reaction for Vimentin (Vim) with CD34 (+), myosin (Desmin) (+), Ki-67 (+, 1%), ER and PR (+), smooth actin (SMA) (−), S-100 negative, and actin (-). The diagnosis according to the immunohistochemistry was CAF.

The patient was discharged on the day after the surgery. MR examination of pelvis performed 2 weeks after the operation showed no abnormality. Follow-up examination 1 month after the operation showed no signs of recurrence.

## 6. Discussion

CAF was first described by Nucci et al^[16]^ in the vulva of middle-aged women in 1997. CAF mainly occurs in the vulva-vaginal area of women with the clinical manifestations of asymptomatic, slowly-growing soft tissue masses, sometimes accompanied by mild pain and discomfort. In the published literature, most articles pertaining to vaginal CAF are case reports or small case series.^[14,17–22]^ Our patient had a well-defined solid and round mass with a maximum length of 2.5 cm, which was consistent with the previously reported cases.

The cause of CAF is still unclear. Lane et al mentioned that long-term use of estrogen therapy may be related to the occurrence of CAF.^[10]^ According to Laskin et al, the extravasation of red blood cells and formation of micro-thrombi in tumors are related to hemodynamic changes. In other words, the tumor cells may originate from differentiation of stem cells around the vascular endothelium into muscle fibers.^[17]^ Our patient had received tamoxifen for more than 5 years after breast cancer surgery. Therefore, it is possible that CAF occurred due to her use of estrogen and was hormone-dependent, which is consistent with related literature reports.

Because vaginal CAFs are less frequent than vulvar CAFs and there are no specific clinical manifestations or signs, it is liable to be misdiagnosed as other vaginal diseases before surgery. For example, in the present case, the lesion was misdiagnosed as a vaginal wall cyst by pelvic examination. In addition, preoperative ultrasound examination did not reveal any vaginal mass, which may be due to the relatively small size of the tumor and the low sensitivity of ultrasound for detection of pelvic mass. Besides, MRI was not performed because of the intrauterine contraceptive device. Therefore, in practice, it is difficult to diagnose this lesion based on preoperative imaging examination.

The definitive diagnosis of CAF can only be made by pathological examination of surgical specimen. Microscopically, the CAF tumor tissues are composed of spindle stromal cells and abundant blood vessels.^[18,23]^ The density of the CAF tumor cells is not uniform and the cells have small nucleus with rare mitotic figures; large multinucleated cells may be seen occasionally. In addition, CAF is rich in small and medium-size blood vessels with thickened and hyaline walls and the interstitium has scattered collagen cellulose of varying thickness, which may show myxoid degeneration and edema.^[18]^ Immunohistochemistry findings are important for the diagnosis of CAF.^[18]^ CAF tumor cells exhibit diffusive and strong positivity for Vim, diffusive positivity for CD34, partial positivity for smooth actin (SMA), and negativity for Desmin (S-100).^[24]^ The pathological features and immunohistochemistry of the present case were consistent with previous reports.

In the majority of the reported CAF cases, there was no recurrence after local simple resection. However, Mc Cluggage et al reported a case of recurrence after resection with negative margins, and Tang et al reported a case of suspected CAF recurrence.^[11,25]^ Therefore, patients with CAF require long-term follow-up after surgery.

Several tumors need to be considered in the differential diagnosis of CAF. First is the invasive angiomyxoma (AAM). AAM is a malignant tumor with large size (usually >5 cm in diameter) located mainly in the pelvic cavity.^[26]^ The boundary of AAM is not well demarcated, and it shows infiltrative growth; moreover, it shows a tendency for recurrence after local excision. On pathological examination, the tumor cells are sparse, and the mucous interstitium is diffuse. In addition, AAM tumor cells are small, uniformly distributed monomorphic spindle-shaped or stellate cells scattered in the lesion, forming good smooth muscle-like cells.^[26]^ On the contrary, CAF is a benign tumor with small size (generally <3 cm in diameter), shallow in site, and typically does not recur after surgical resection. AAM is typically positive for vimentin and myosin, while CAF is typically diffusively and strongly positive for vimentin (Vim), CD34(+), and S-100 (−). Another tumor that may mimic CAF is vaginal angiomyofibroblastoma. AMF shows a clear boundary under the microscope, with large parenchymal blood vessels of variable sizes, distributed in clusters, and diversified tumor cells and nuclei. In addition, the myofibroblasts are unevenly distributed, with densely packed areas of multiple cells alternating with regions of low cellular density. However, the tumor cells in the dense area show perivascular arrangement, which are distributed in the myxedematous interstitium, with no cellular atypia.^[19,27]^ Under high magnification, there is extensive distribution of collagen fibers between the tumor cells. Besides, the hypocellular areas show mucin-edema-like background and the cells are long fusiform with slender cytoplasm, deeply stained nuclei, and no mitoses.^[28]^ On immunohistochemistry examination, AMF cells are typically diffusely and strongly positive for sarcomeric protein, but negative for CD34, S-100 cytokeratin, and epithelial membrane antigen.^[23]^ Last but not the least, is the vaginal solitary fibrous tumor, which is composed of spindle cells and abundant blood vessels, and the immune tissue marker CD34 is always positive.^[29,30]^ On the contrary, CAF tumor cells exhibit diffusive and strong positivity for Vim, diffusive positivity for CD34, partial positivity for smooth actin (SMA), negativity for Desmin (S-100).^[24]^

Owing to the rarity of CAF, the present case report adds value to the contemporary literature by describing the clinicopathological characteristics of this disease. However, the short follow-up period in this case report is a limitation.

## 7. Conclusion

In summary, CAF is a rare benign soft tissue tumor, which is difficult to diagnose before surgery. Surgery is the only treatment method, and definitive diagnosis of CAF is made by histopathological examination of surgical specimen. Long-term follow-up is needed for surveillance of recurrence.

## Author contributions

**Conceptualization:** Zeng-Li Shen, Ya-Zhen Zhang.

**Investigation:** Xia-Qin Cai, Xi-Gang Ye, Ke Hong.

**Supervision:** Shu-Zhi Zhang.

**Validation:** Shu-Zhi Zhang, Xia-Qin Cai.

**Visualization:** Zeng-Li Shen.

**Writing – original draft:** Xia-Qin Cai, Zeng-Li, Ke Hong. Shu-Zhi Zhang.

**Writing – review & editing:** Xia-Qin Cai, Ke Hong.
